# KASP Markers Specific for the Fertility Restorer Locus *Rf1* and Application for Genetic Purity Testing in Sunflowers (*Helianthus annuus* L.)

**DOI:** 10.3390/genes13030465

**Published:** 2022-03-05

**Authors:** Aleksandra Radanović, Yves Sprycha, Milan Jocković, Monja Sundt, Dragana Miladinović, Constantin Jansen, Renate Horn

**Affiliations:** 1 Institute of Field and Vegetable Crops, Maksima Gorkog 30, 21000 Novi Sad, Serbia; aleksandra.dimitrijevic@ifvcns.ns.ac.rs (A.R.); milan.jockovic@ifvcns.ns.ac.rs (M.J.); dragana.miladinovic@ifvcns.ns.ac.rs (D.M.); 2 Department of Plant Genetics, Institute of Biological Sciences, University of Rostock, Albert-Einstein-Str. 3, D-18059 Rostock, Germany; yves.sprycha@outlook.de (Y.S.); monja.sundt@uni-rostock.de (M.S.); 3 Strube Research GmbH & Co. KG, Hauptstr. 1, D-38387 Söllingen, Germany; constantin.jansen@graminor.no

**Keywords:** candidate gene, EMS mutagenesis, genetic purity, hybrid breeding, KASP marker, marker-assisted selection, CMS PET1 cytoplasm, restorer gene *Rf1*, single nucleotide polymorphism, sunflower

## Abstract

Single nucleotide polymorphisms (SNPs) were significantly associated with fertility restoration of cytoplasmic male sterility (CMS) PET1 by the restorer gene *Rf1*. For these SNPs, four Kompetitive allele-specific PCR (KASP) markers were successfully designed. The KASP markers cover the fertility restorer locus *Rf1*, spanning about 3 Mb, and clearly differentiate restorer and maintainer lines. For genetic purity testing in sunflower hybrid production, the efficiency for detecting contaminations in samples was simulated using mixtures of hypocotyls or leaves. Contaminations of restorer lines with 1%, 3%, 5%, 10%, and 50% of maintainer lines were screened with all four KASP markers. Contaminations of 10% could be clearly detected in pools of 100 plants. Contaminations below this level require detection on a single plant level. For single plant detections, ethyl methanesulfonate-treated sunflower F1 hybrids, which had been phenotypically evaluated for male sterility (potential mutation in the *Rf1* gene) were screened. Nine identified either partially male-sterile or male-sterile plants were analyzed with all four KASP markers and only one proved to be a hybrid with a mutation, seven were male-sterile contaminants in the F1 seeds used (1.6%) and one a recombinant plant. The four KASP markers should be valuable tools for marker-assisted selection (MAS) in sunflower breeding regarding the restorer locus *Rf1*.

## 1. Introduction

Sunflower breeding is based on hybrid production, which exploits heterosis, the phenotypic superiority of a hybrid over its parents. Hybrids are obtained by crossing male sterile (A) lines with restorer (R) lines. In sunflowers, the most commonly used cytoplasmic male sterility source is the CMS-PET1 cytoplasm [1]. The maternally transmitted trait is manifested in an inability to produce pollen due to rearrangements of the mitochondrial genome [2,3]. For the maintenance of A lines, fertile analogs, called maintainer or B lines, which carry a normal, fertile cytoplasm, are necessary.

Restoration of male fertility in the F1 hybrids requires dominantly inherited nuclear restorer-of-fertility (*Rf*) genes. Despite the availability of several *Rf* genes such as, e.g., *Rf3* or *Rf5*, *Rf1* has been the most widely exploited restorer gene in sunflower hybrid breeding for over four decades [4,5]. The fertility restorer gene *Rf1* was introduced into sunflower breeding material from the T66006-2-1-B line [6]. The genetic nature of inheritance of *Rf1* makes this trait very eligible for detection by use of molecular markers. In the past, *Rf1* was positioned on linkage group (LG) 13 of the sunflower genetic reference map [7]. Different types of markers, such as restriction fragment length polymorphism (RFLP), random-amplified polymorphism DNA (RAPD), amplified fragment length polymorphism (AFLP), sequence characterized amplified region (SCAR), target region amplification polymorphism (TRAP), simple-sequence repeats (SSR), and cleaved amplified polymorphic sequences (CAPS) have been used for mapping the gene [7,8,9,10]. Advancements in technology and the availability of the sunflower reference genome [11] have enabled a more detailed analysis of the locus comprising the *Rf1* gene. However, the *Rf1* gene itself has not been identified mainly due to the complexity and size of the sunflower genome, represented by the maintainer line HanXRQ.

Recently, candidate genes for *Rf1* have been identified using SNP-based association studies. Goryunov et al. [12] applied a genome-wide association study and found 21 candidate genes for *Rf1* in a defined segment spanning 7.72 Mb on LG 13. Twenty of these genes belong to the pentatricopeptide repeat (PPR) gene family and one additional gene was annotated as a probable aldehyde dehydrogenase gene [13]. Talukder et al. [5] associated 24 SNP markers spanning 2.47 cM on LG 13 with fertility restoration by genome-wide association studies (GWAS) using a mixed model analysis in a panel of 333 sunflower lines. Further studies demonstrated that 130 of the 548 examined lines had retained all 24 SNPs associated with *Rf1* indicating an inheritance of all SNPs as one haplotype throughout decades of sunflower breeding [5]. However, these 24 SNPs were also present in three maintainer lines released by the Agricultural Research Service (ARS) of the United States Department of Agriculture (USDA). Finally, Horn et al. [14] were able to narrow down the number of candidate genes for fertility restoration to three candidate genes belonging to the PPR gene family (HanXRQChr13g0419621, HanXRQChr13g0418841, and HanXRQChr13g0418861). These three genes were identified by 10 SNPs significantly associated with fertility restoration in an association study using 32 restorer and 27 maintainer lines. For this purpose, amplicon-targeted sequencing of nine potential candidate genes was used to obtain the gene sequences for the SNP analyses. Three SNP-based markers, one co-dominant PAMSA (polymerase chain reaction (PCR) amplification of multiple specific alleles), and two dominant markers were developed, which clearly differentiate restorer from maintainer lines [14]. All three gel-based markers were verified in an association panel consisting of 557 sunflower accessions.

Polivanova et al. [15] found significant variability in the *Rf1* locus (position 169,655,088 to 178,217,103, HanXRQr1.0 annotation) by using PCR gene-specific primer pairs for the previously investigated 25 potential candidate genes for *Rf1* [12,14].

Real-time PCR markers for high-throughput detection of the *Rf1* gene would greatly facilitate and add to the cost-effectiveness in hybrid production, especially in sunflowers, the second most important crop based on hybrid breeding [16]. Assessing the genetic purity of the hybrid components represents an essential quality control function in hybrid production. The genetic purity of the seeds can be compromised by out-crossing with foreign pollen, but also by seed contaminations [17,18]. Several approaches are applied to analyze genetic purity: (1) grow out test (GOT), (2) use of biochemical, and (3) molecular markers. While the first approach is based on detecting morphological differences, the second and the third are oriented towards detecting protein/isoenzyme and DNA profiles, respectively. From the three techniques, molecular markers are the most precise, especially if co-dominantly inherited. First, RAPD markers were used directly [19] or converted into SCARs for analyzing hybrid purity [20]. To this day, the most commonly used type of markers for genetic purity assessment are SSRs, which are co-dominant and highly polymorphic. In addition, other sets of markers have been used to distinguish inbred lines [17,21,22]. However, none of the mentioned marker techniques are as prone to automatization, high-throughput, and cost-effectiveness as the new real-time marker platforms developed for the detection of SNPs, such as TaqMan, KASP, high-resolution melting (HRM), and rhAmp. KASP is a single-step genotyping technology based on an end-point real-time PCR amplification coupled with fluorescence detection [23]. KASP assays have already been successfully used for the detection of *Rf* genes in other crop species such as sorghum [24] and pepper [25]. In rice, KASPs were exploited for the detection of two thermo-sensitive genic male sterility genes (*p/tms12-1* and *tms9-1*) in analyses for seed purity [26]. In wheat, KASP assays showed 45 times higher superiority in speed over classical gel-based markers [27].

In this study, four KASP markers were developed for the detection of SNPs significantly associated with the three potential candidate genes for the *Rf1* gene in sunflowers [14]. KASP assays were tested in a selected panel of restorer lines, maintainer lines, and hybrids in order to access their potential use in marker-assisted selection (MAS). Furthermore, the potential sensitivity of the developed markers in genetic purity testing in hybrid breeding was investigated by analyzing mixtures representing different levels of contaminations. In addition, single plant detections of ethyl methanesulfonate-treated (EMS) sunflower F1-hybrid plants, which had been phenotypically screened for male sterility, were performed.

## 2. Materials and Methods

### 2.1. Sunflower Plant Material

Nine maintainer lines and nine restorer lines as well as 18 hybrids of these lines were used to validate the KASP markers. The hybrids were obtained by either crossing the maintainer lines with a commercial restorer tester or by pollinating a commercial CMS-tester with the restorer lines of the panel (Table S1). Male fertility of the F1 hybrids was confirmed in the field at Bandow/Schwaan (Germany) in 2019. In addition, three other maintainer and five other restorer lines of the PET1 cytoplasm were included in the study.

For simulation of contaminations in inbred lines, seedlings were grown in a growth chamber for three weeks and pools of 100 hypocotyl pieces (1 cm) were generated representing 1%, 3%, 5%, 10%, and 50% contamination (Table S2). For simulation of contaminations, different amounts of two maintainer lines (UGA-SAM1-109 or UGA-SAM1-082) were added to two restorer lines (UGA_SAM1-136 or UGA-SAM1-191). In addition, leaf discs (Ø 1cm) were cut from 3-weeks-old seedlings (Table S3). Contaminations of 1%, 3%, 5%, 10%, and 50% were investigated in comparison to both pure lines and four hybrids (LC × UGA-SAM1-136, LC × UGA-SAM1-109, UGA-SAM1-109 × R, UGA-SAM1-082 × R).

### 2.2. EMS-Treatment of Sunflower Seeds

A total of 1040 F1-hybrid (cmsHA342 × RHA325) seeds representing combined seed samples were treated with 0.8% or 1.2% ethyl methanesulfonate. For the mutagenesis treatment, seeds were pre-germinated for 24 h in 0.1% KCl by slightly shaking them. After this period, the seeds were incubated with two different EMS concentrations (0.8% or 1.2%) for 18 h. Finally, the seeds were washed three times with sodium thiosulfate to inactivate the toxic EMS. Seeds were immediately planted on the field and watered to secure germination. Germination rates were 41% (213/520) for the 0.8% EMS treatment and 41.9% (218/520) for 1.2% EMS. Plants were scored for fertility in the field in 2019 at Bandow/Schwaan (Germany). Leaves were taken and frozen at −20 °C for DNA analysis.

### 2.3. DNA Extraction

DNA was extracted from sunflower leaves harvested from the restorer and maintainer lines as well as the F1 hybrids in the field and stored at −20 °C. For the genetic purity testing, hypocotyl segments of sunflower seedlings (1 cm) were used that had been pooled according to the contamination protocol before being frozen in liquid nitrogen and stored at −20 °C. Prior to DNA extraction, the pooled samples were homogenized using a pestle and mortar. The DNA was isolated by the CTAB protocol of Doyle and Doyle [28] starting with 200–400 mg leaf material or hypocotyls. The final DNA pellet was dissolved in TE buffer (1 mM EDTA, 10 mM Tris HCl pH 8.0) and diluted with sterile ddH_2_O to 15 ng/μL for KASP assays.

### 2.4. SNP Analyses Using Amplicon Targeted Sequences

Ten maintainer lines and 10 restorer lines, used for validation of the KASP assays, belonged to the 59 sunflower accessions that were part of the amplicon targeted sequencing of nine potential candidate genes for *Rf1* by LGC Genomics, Berlin, Germany [14]. Applying the Ovation Custom Target Enrichment System (NuGen Technologies, Tecan Group Ltd., Männedorf, Switzerland) library enrichment was performed, followed by sequencing on the MiSeq V3 subunit (2 × 300 bp) with 120× coverage. Variant calls were executed by LGC Genomics and supplied in VCF files.

### 2.5. KASP Primer Design

KASP markers were designed for four SNPs significantly associated with fertility restoration of CMS PET1 by the restorer gene *Rf1* [14]. Positions of the SNPs and the three genes are given in the new sunflower genome sequence assembly in comparison to the old assembly in Table S4**.** The sequences around the SNPs are shown in Table S5. The PrimerQuest tool with the default setting of qPCR 2 Primers Intercalating Dyes (Primers only) (https://www.idtdna.com/SciTools, last accessed on 3 March 2022) was used to design KASP primers (Table 1).

For the development of the KASP markers, the length of the PCR products was restricted to <150 bp and an annealing temperature close to 62 °C was chosen. Each primer combination consists of two allele-specific primers with unique tails at the 5′-end corresponding to the universal FRET (fluorescence resonant energy transfer) cassette for each fluorescent dye (FAM: 5′-GAAGGTGACCAAGTTCATGCT-3′ and HEX: 5′-GAAGGTCGGAGTCAACGGATT-3′) and one common primer (Table 1). Primers were ordered from Biolegio (Nijmegen, Netherlands) and dissolved in TE buffer (1 mM EDTA, 10 mM Tris HCl pH 8.0). The primer mix for a single reaction contained 0.01 μL of each allele-specific primer (100 pmol/μL) and 0.3 μL of the common primer (100 pmol/μL) added up to 0.1 μL with 0.05 μL ddH_2_O.

### 2.6. KASP-Marker Assay

For the KASP assay, a slightly modified protocol according to Patterson et al. [29] was used. The assays were run on a LightCycler 96 (Roche Diagnostic, Mannheim, Germany). Each reaction (8 μL) contained 4 μL DNA (15 ng/μL) or 4 μL ddH_2_O as a negative control, 0.1 μL primer mix and 3.9 μL KASP master mix (LGC Genomics, Berlin, Germany) containing the universal FRET cassettes for FAM and HEX labeled oligonucleotides, low ROX passive reference dye, Taq polymerase, dNTPs, buffer with optimized MgCl_2_ concentration. The PCR program began with a hot start activation at 94 °C for 900 s, followed by 10 cycles of touchdown PCR (denaturation: 94 °C for 20 s, polymerization: starting at 61 °C with a sequential reduction in the temperature by 0.6 °C to 55 °C), followed by 26 cycles (denaturation: 94 °C for 20 s, polymerization: 55 °C for 60 s) and 2 cycles 37 °C for 60 s, 37 °C for 10 s. Results of the PCR were determined as end-point readings after lowering the temperature to 37 °C.

### 2.7. Data Analysis

For the data analysis the measured fluorescence values for FAM and HEX were transformed into percentages of fluorescence (% F) by adjusting them to the corresponding minimum and maximum fluorescence values for each fluorescence dye on each measured plate, respectively. This has to be performed separately for each fluorescence dye, either Hex or Fam. The following modified formula according to Oliveira et al. [30] was used:

% F = (X − min. F/max. F − min. F) × 100 (1)

where X represents the measured fluorescence of the individual dye, either Hex or Fam, min. F is the lowest fluorescence value (minimum) measured on the 96-well plate for the respective dye and max. F is the highest fluorescence value of the respective dye (maximum) for this plate measurement. A no amplification zone was defined below 25% for the fluorescence values, as the data points of the negative controls carrying no template or assays not amplifying were located in this area. Two replications per sample were sufficient for the differentiation between maintainer lines, restorer lines, and hybrids, but four replications per sample were applied for the contamination testing and the screening of the EMS mutants. The dashed lines represent the borders for scoring into the different classes (restorer, hybrid, maintainer). In the ideal case as for KASP 621.5, the two dashed lines separate the 90 degrees into three 30-degree segments.

## 3. Results

### 3.1. Development of KASP Markers Based on SNPs Associated with Fertility Restoration

**Figure 1:**
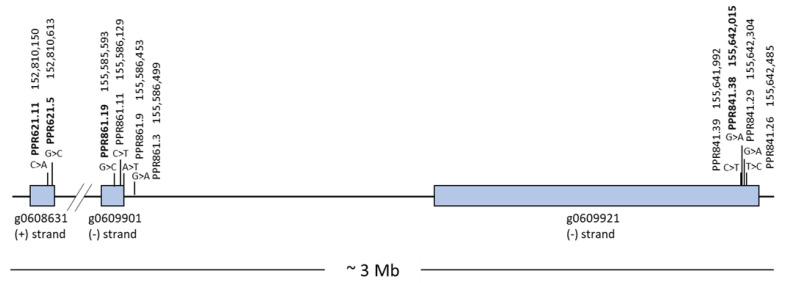
Locations of SNPs significantly associated with the fertility restorer locus *Rf1* in the new sunflower genome assembly HanXRQr2. SNPs successfully used for KASP marker design are shown in bold.

Starting with 10 SNPs, which had been significantly associated with fertility restoration of the PET1 cytoplasm [14], four SNPs (PPR621.5, G > C; PPR621.11, C > A; PPR841.38, G > A; and PPR861.19, G > C) were selected for the development of KASP markers because these were reported to be homozygous in the restorer lines. Locations of all 10 SNPs in the new sunflower genome assembly HanXRQv2 are given in Figure 1. For all selected SNPs, KASP markers could be developed.

The four KASP markers (621.5, 621.11, 841.38 and 861.19) represent the three potential gene candidates HanXRQr2_Chr13g0608631, HanXRQr2_Chr13g0609921, and HanXRQr2_Chr13g0609901 at the restorer locus *Rf1*, spanning a genome region of about 3 Mb in the new whole-genome assembly HanXRQr2. The KASP markers were first validated with regard to their ability to differentiate between nine restorer lines, nine maintainer lines, and 18 F1 hybrids. In addition, three other maintainer and five additional restorer lines were included (Table S1).

**Figure 2:**
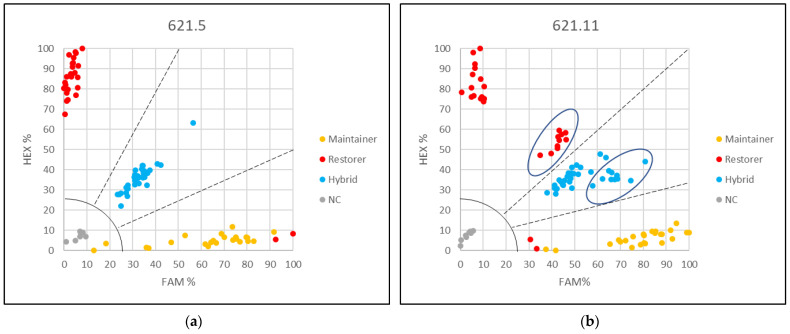
Grouping obtained by KASP markers (**a**) 621.5 and (**b**) 621.11. The latter splits the restorer lines into two groups. However, the outgroup of restorer lines (marked by an oval) can still be separated from their crosses to the CMS tester (also marked by an oval). NC negative control.

The potential restorer gene HanXRQr2_Chr13g0608631 (PPR621) is represented by two KASP markers for the SNPs PPR621.5 and PPR621.11. The first one, KASP marker 621.5, gave the best separation between restorer lines, maintainer lines, and F1 hybrids (Figure 2a). Interestingly, one of the tested restorer lines, IH-51, carries the PET1 cytoplasm and is not only a restorer line for PET1 but also a restorer line for the PET2 cytoplasm [31], behaved differently than expected. IH-51 was clearly grouped with the maintainer lines indicating the absence of *Rf1* in IH-51 despite its restoration ability towards PET1.

In the differentiation of restorer lines, maintainer lines, and hybrids, KASP marker 621.11 gave a slightly different picture compared to 621.5 as it splits the restorer lines into two groups (Figure 2b). Five of the restorer lines (UGA-SAM1-010, UGA-SAM1-024, UGA-SAM1-136, UGA-SAM1-161, and UGA-SAM1-191) formed a separate group closer to the F1 hybrids, but their hybrids with a commercial CMS tester tended also to be more located towards the maintainers still allowing a clear differentiation between restorer lines, maintainer lines, and F1 hybrids.

**Figure 3:**
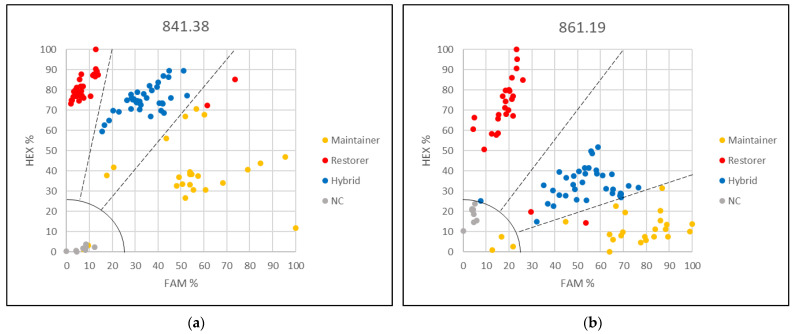
Grouping obtained by KASP markers (**a**) 841.38 and (**b**) 861.19. NC negative control.

The two KASP markers for detecting the SNPs PPR841.38 and PPR861.19 located in the two other potential restorer candidate genes (HanXRQr2_Chr13g0609921 and HanXRQr2_Chr13g0609901, respectively) also gave a clear differentiation, but to a lesser degree (Figure 3). Using these two KASP markers in addition to 621.5 and 621.11 allows coverage of the whole restorer locus *Rf1* of about 3 Mb on LG 13 for analyses of recombination events.

Specific SNP detection by the developed KASP markers was validated by comparison to the SNP analyses performed for 10 of the maintainer lines and 10 of the restorer lines by amplicon targeted sequencing (Table 2). The nucleotides were confirmed correct in all cases, but samples of CM63 fell into the 25% no amplification zone for KASP 841.38 and 861.19. For KASP marker 841.38, UGA-SAM1-109 indicated a heterozygous state.

### 3.2. Applying the KASP Marker 621.5 for Genetic Purity Testing

**Figure 4:**
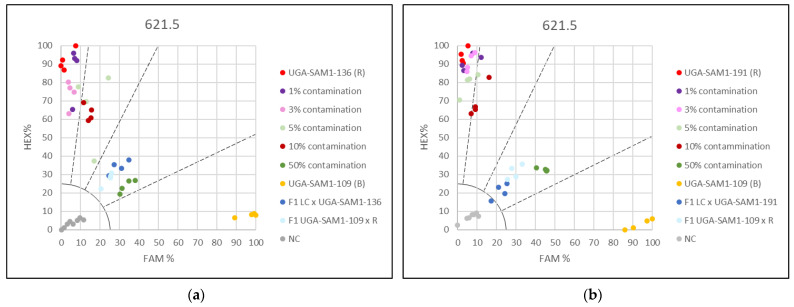
Genetic purity testing for hybrid breeding using hypocotyls and KASP marker 621.5. (**a**) Contaminations of restorer UGA-SAM1-136 with maintainer UGA-SAM1-109 were simulated and (**b**) Contaminations of restorer UGA-SAM1-191 with maintainer UGA-SAM1-109.

Genetic purity testing in sunflower hybrid production requires SNP-based markers for high-throughput automated screening of breeding material. Sensitivity of the KASP marker 621.5, which provided the best separation results, was analyzed in mixtures representing different levels of contaminations (Tables S2 and S3). In addition, reference samples representing the pure restorer line, the pure maintainer line, and the hybrids were included in the KASP assays. Two types of plant tissue, hypocotyls and leaves, were compared for their efficiency in contamination screening. To show the universal use of the KASP marker in genetic purity testing, two different sets of mixtures per plant tissue type were created. In the KASP assays based on hypocotyl mixtures, 1%, 3%, 5%, and 10% contaminations grouped together with the pure restorer line sample. However, it was notable that the pure restorer line and less contaminated samples (1% and 3%) were closer to the 100% HEX axis compared to samples with 5% and 10% contamination (Figure 4). A clear distinction was observed only in the case of 50% contamination, compared to the group of 0–10% contamination. The maintainer line was clearly separated from all other samples.

**Figure 5:**
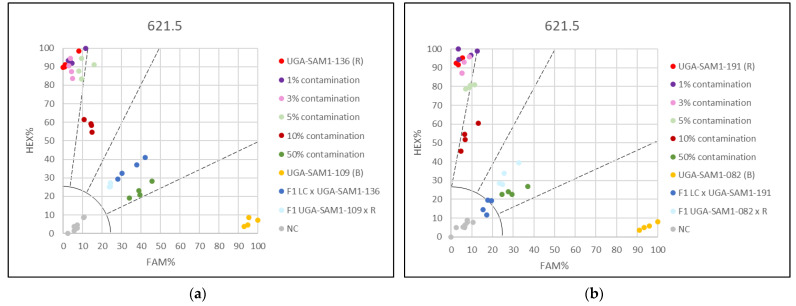
Genetic purity for hybrid breeding using leaves and KASP marker 621.5. (**a**) Contaminations of restorer UGA-SAM1-136 with maintainer UGA-SAM1-136 were simulated and (**b**) Contaminations of restorer UGA-SAM1-191 with maintainer UGA-SAM1-082.

Better results were observed when leaf mixtures were used. Mixtures with 1%, 3%, and 5% contamination grouped together with the pure restorer (Figure 5), but the mixture representing 10% contamination was clearly separated from this group as well as from the sample with 50% contamination. In all KASP assays, the mixture representing 50% contamination grouped together with the F1 hybrids as expected (Figure 4 and Figure 5). Maintainer lines arranged separately from all other samples. Results obtained from different genotype sets, tested on the same tissue type, were comparable. Contaminations below 10% will require single plant analyses in order to estimate the percentage of contamination.

### 3.3. Applying the KASP Markers for Single Plant Testing

**Figure 6:**
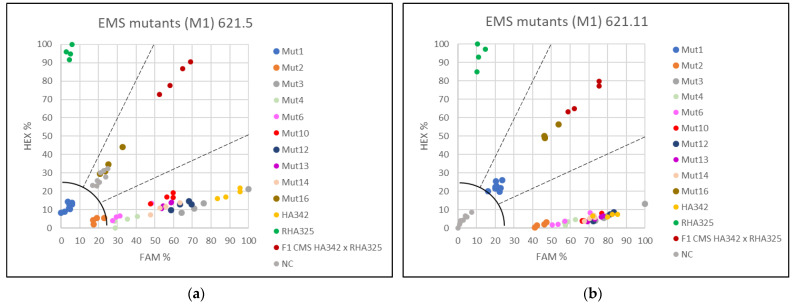
Investigation of the restorer locus *Rf1* in EMS-treated plants (M1 generation) by application of two KASP markers. (**a**) Screening with KASP 621.5, (**b**) Screening with KASP 621.11.

**Figure 7:**
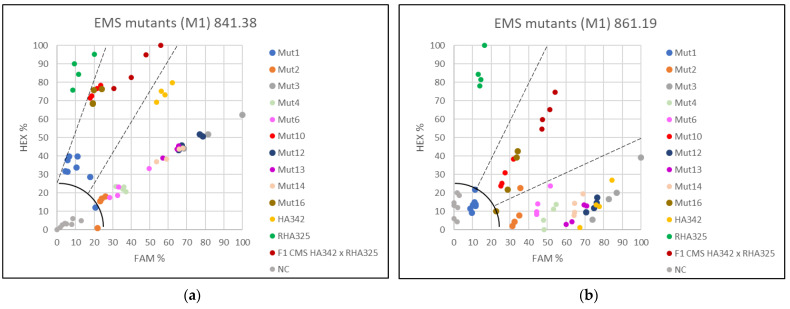
Investigation of the restorer locus *Rf1* in EMS-treated plants (M1 generation) by application of two KASP markers. (**a**) Screening with KASP 841.38 and (**b**) Screening with KASP 861.19.

F1-hybrid seeds (cmsHA342 × RHA325) were treated with 0.8% or 1.2% EMS to obtain male sterile plants with mutations in the restorer gene *Rf1*. Investigating 213 sunflower plants treated with 0.8% EMS, no male-sterile plant was observed. However, using 1.2% EMS, nine out of 218 germinated plants were partially male sterile or male sterile. For verification of the plant origin, these nine male-sterile or partially male-sterile EMS-treated F1 plants, and one male fertile EMS F1 mutant with basal branching as an internal reference, were analyzed using all four KASP markers 621.5 and 621.11 (Figure 6) and 841.38 and 861.19 (Figure 7).

For KASP markers 621.11, 841.38 and 861.19 partially male-sterile mutant Mut1 clearly grouped with the F1 hybrid cmsHA342 × RHA325 (*Rf1rf1*). KASP marker PPR621.5 showed no amplification for Mut1. The F1 hybrid character of the male-fertile branching mutant Mut16 was also confirmed by all four KASP markers.

Seven male-sterile EMS mutants grouped together with the maintainer line HA342 (*rf1rf1*) with all four KASP markers indicating that they do not contain a dominant restorer allele *Rf1*, neither normal nor mutated by EMS. Mutant Mut10 showed a different behavior for the two PPR621-derived KASP markers than for the other two markers. For PPR621, both KASP markers (621.5 and 621.11) grouped Mut10 with the maintainer HA342, explaining the observed partial male sterility. For the KASP markers 841.38 and 861.19, Mut 10 grouped with the F1 hybrid cmsHA342 x RHA325 detecting a recombination event at the restorer locus *Rf1* between these two SNPs and the two KASP markers for PPR621. As Mut10 was partially male sterile, this recombination indicates that HanXRQr2_Chr13g0608631 (PPR621) is more important for fertility restoration of PET1 than the other two candidate genes. Single plant KASP assays allowed classifying each plant into a group, either maintainer (*rf1rf1*), restorer (*Rf1Rf1*), or F1 hybrid (*Rf1rf1*).

**Figure 8:**
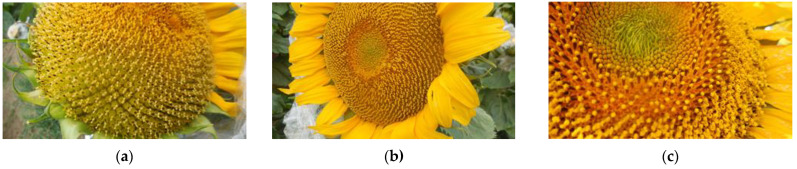
Phenotypic evaluation of the partially male sterile EMS-treated F1 plants Mut 1 (**a**) und Mut 10 (**b**) in comparison to the male fertile, untreated F1 hybrid cmsHA342 × RHA325 (**c**).

The KASP assays showed that only the partially male-sterile Mut1 and the male-fertile EMS-branching mutant Mut16 proved to be F1 hybrids, whereas Mut10 represents a male-sterile recombinant plant, where a recombination event had occurred in the region between HanXRQr2_Chr13g0608631 (PPR621) and the two other genes HanXRQr2_Chr13g0609921 (PPR841) and HanXRQr2_Chr13g0609901 (PPR861) (Figure 8).

## 4. Discussion

In this study, KASP markers were exploited for the detection of *Rf1*-associated SNPs and tested for their efficiency in genetic purity assessment. KASP assays offer the possibility of automatization, high-throughput application, and good cost-effectiveness. All of these properties are important in both MAS and genetic purity testing as both processes involve the analysis of thousands of plants.

All four developed KASP markers (621.5, 621.11, 841.38 and 861.19) enabled discrimination between restorer lines, maintainer lines, and hybrids. KASP markers developed for the detection of two SNPs within the gene HanXRQr2_Chr13g0608631 (PPR621) had higher discrimination power compared to the KASP markers developed for the detection of the two SNPs identified in the genes HanXRQr2_Chr13g0609921 (PPR841) and HanXRQr2_Chr13g0609901 (PPR861), respectively. Even though there is a large number of reports of successful conversions of SNPs to KASP assays, not all SNPs can be successfully used to design KASP markers, because additional SNPs in the primer regions or within the amplified regions may interfere with design and results [27,32,33]. The sequence of the *Rf1* gene may be complex and its structure may vary [15], thus it can be expected that not all KASP assays developed will be equally efficient in discrimination between restorer and maintainer lines. The developed KASP markers were tested across a panel of sunflower genotypes composed of restorer and maintainer lines, and hybrids. The nature of all tested genotypes was confirmed by all four KASP assays, with the exception of restorer line IH-51, which grouped with the maintainer line. This line is a restorer line for both PET1 and PET2 cytoplasm [31], and obviously does not possess the *Rf1* gene, but the *Rf-PET2* gene that is positioned close to *Rf1*, mapping on the lower part of LG 13 [34]. Consequently, fertility restoration of PET1 in IH-51 must occur via a different restorer gene. KASP markers developed in this study were efficient enough to enable differentiation between restorer lines with different but closely mapped *Rf* genes, thus proving their discriminative power.

KASP marker 621.5 enabled the most efficient discrimination between the tested genotypes and was therefore chosen for further validation in the genetic purity testing for hybrid production. As the sensitivity of a genetic purity test is determined by the separation strength between pure and contaminated samples [35], two line sets with different contamination levels were tested. Moreover, two different tissue types were used, leaves and hypocotyl, as the efficiency of tissue disruption will have an influence on the results. Leaf tissue enabled better discrimination between the samples. The reason could lie in better tissue disruption of plant leaves compared to hypocotyls, as frozen hypocotyls were harder to be mechanically pulverized. When using leaf tissue as a DNA source, samples with 10% contamination were clearly separated from samples with lower percentages of contamination, which was not the case when hypocotyls were used. In addition, using leaf tissue has the advantage that the whole plant does not need to be destroyed for testing.

In some cases, individual plants need to be analyzed in order to increase precision in detecting contaminations. This is particularly important in seed production, where minimum varietal purity of sunflower hybrids of 95% is required, while 99.5% and 99.8% are requested for female and male line production, respectively (OECD Seed Scheme, 2021 [36]). So far, only gel-based molecular markers have been used for genetic purity screening in sunflowers [17,19,20,21], but they are not as prone to automatization as KASP markers. Furthermore, KASP assays have proven to be very cost effective compared to other technologies used for the detection of SNPs, as in one study this technique was calculated to be 2.6 times cheaper than TaqMan assays [37]. Yuan et al. [38] even estimated that the cost per data point is only 1/50 using KASP assays compared to SNP genotyping by TaqMan. As equipment, only an RT-PCR cycler is necessary for KASPs. Taking advantage of the high-throughput possibilities of KASP assays, the detection of contamination by analysis of individual plants has been successfully performed in other plant species such as rice [26] and maize [39,40].

In our work, single plant testing was performed on phenotyped male-sterile or partially male-sterile plants obtained by EMS treatment of F1 hybrids (cmsHA342 × RHA325). For the EMS treatment, different seed charges had been combined to obtain enough seeds. All four markers clearly grouped the analyzed plants in one of the three categories (*Rf1Rf1*, *Rf1rf1*, *rf1rf1*). As the four KASP markers developed in this study cover the entire *Rf1* restorer locus, it was also possible to detect a recombination event that occurred in one F1 plant. Mut 10 showed the genetic constitution of the maintainer (*rf1rf1*) for KASP marker 621.5 and 621.11, but for KASP 841.38 and 861.19, it was heterozygous (*Rf1rf1*). This detected recombination event might be responsible for the male-sterile phenotype in Mut 10. This must be further verified by additional molecular studies. Furthermore, the KASP marker analysis clearly demonstrated that seven of the potential EMS mutants were indeed contaminants (1.6%) and did not represent F1-hybrid mutants. The easiest explanation for these male-sterile plants is that one smaller seed charge did not represent F1 hybrids, but accidentally F2 seeds obtained by selfing of F1 hybrids. An F2 population would segregate into fertile and male-sterile plants in a ratio of 3:1. This is also supported by the fact that some of the male-sterile lines were branched. Branching is a recessive trait coming from the restorer line RHA325 used in the cross combination.

Single plant testing demonstrated the efficiency of the four KAPS markers in identification of possible contaminations and recombination events. It also showed that the molecular characterization of the M1 generations plants is essential in an EMS mutagenesis trial.

## 5. Conclusions

All four KASP markers (621.5, 621.11, 841.38 and 861.19) identify the restorer gene haplotype of *Rf1* and can be used to select for this restorer locus. KASP 621.5 proved to be the most reliable marker with the best discrimination between maintainer lines, restorer lines, and hybrids. Using KASP marker 621.5 for genetic purity testing showed that contaminations of 10% can be detected in mixtures containing leaves. For a lower percentage of contaminations, single plant assays have to be performed. The four KASP markers prove to be a universal tool to detect recombination events at the restorer locus *Rf1*.

## Figures and Tables

**Table 1:** KASP primers designed for the SNPs PPR621.5, PPRR621.11, PPR841.38, and PPR861.19.

Primer Name	Primer Sequences (5′-3′) ^1^
**PPR621.5 (G/C)**	
621.5 F1_FAM	*GAAGGTGACCAAGTTCATGCT*ACCAGTAATCTCCACATGAACATTG
621.5 F2_HEX	*GAAGGTCGGAGTCAACGGATT*ACCAGTAATCTCCACATGAACATTC
621.5 R1	GCGATAAAGAAGCGGGAGATTA
**PPR621.11 (C/A)**	
621.11 F3_FAM	*GAAGGTGACCAAGTTCATGCT*GCGGACGCTTGTATGTTC
621.11 F4_HEX	*GAAGGTCGGAGTCAACGGATT*GCGGACGCTTGTATGTTA
621.11 R3	TACGGGTGGACCCACAT
**PPR841.38 (G/A)**	
841.38 F3_FAM	*GAAGGTGACCAAGTTCATGCT*GCAAAGCACTTGTTTCGTAG
841.38 F4_HEX	*GAAGGTCGGAGTCAACGGATT*GCAAAGCACTTGTTTCGTAA
841.38 R3	ATCCCTGGAGAAGAACATTGT
**PPR861.19 (G/C)**	
861.19 F3_FAM	*GAAGGTGACCAAGTTCATGCT*AAAAGAAATGGAGGAGGATG
861.19 F4_HEX	*GAAGGTCGGAGTCAACGGATT*AAAAGAAATGGAGGAGGATC
861.19 R3	CTTCATGCACCTTACCTTCC

^1^ unique tail corresponding to the individual FRET cassettes is shown in italics.

**Table 2:** Validation of the KASP marker results by comparison with the SNP analyses from amplicon targeted sequencing.

Common Name	Sequence-Based SNP Analyses				KASP Marker ^1^			
	PPR621.5	PPR621.11	PPR841.38	PPR861.19	621.5	621.11	841.38	861.19
Armavirsky 3497	GG	CC	GG	GG	GG	CC	GG	GG
Arrowhead	GG	CC	GG	GG	GG	CC	GG	GG
CM259	GG	CC	GG	GG	GG	CC	GG	GG
CM63	GG	CC	GG	GG	GG	CC	-	-
Krasnodaret	GG	CC	GG	GG	GG	CC	GG	GG
No. 2	GG	CC	GG	GG	GG	CC	GG	GG
UGA-SAM1-082	GG	CC	GG	GG	GG	CC	GG	GG
UGA-SAM1-109	GG	CC	GG	GG	GG	CC	GC	GG
UGA-SAM1-156	GG	CC	GG	GG	GG	CC	GG	GG
UGA-SAM1-185	GG	CC	GG	GG	GG	CC	GG	GG
HA342	-	-	-	-	GG	CC	GG	GG
HA383	-	-	-	-	GG	CC	GG	GG
UGA-SAM1-010	CC	AA	AA	CC	CC	AA	AA	CC
UGA-SAM1-024	CC	AA	AA	CC	CC	AA	AA	CC
UGA-SAM1-100	CC	AA	AA	CC	CC	AA	AA	CC
UGA-SAM1-101	CC	AA	AA	CC	CC	AA	AA	CC
UGA-SAM1-121	CC	AA	AA	CC	CC	AA	AA	CC
UGA-SAM1-136	CC	AA	AA	CC	CC	AA	AA	CC
UGA-SAM1-161	CC	AA	AA	CC	CC	AA	AA	CC
UGA-SAM1-169	CC	AA	AA	CC	CC	AA	AA	CC
UGA-SAM1-191	CC	AA	AA	CC	CC	AA	AA	CC
UGA-SAM1-204	CC	AA	AA	CC	CC	AA	AA	CC
RHA325	-	-	-	-	CC	AA	AA	CC
RHA265	-	-	-	-	CC	AA	AA	CC
IH-51	-	-	-	-	GG	CC	GG	GG
NS-H-27	-	-	-	-	CC	AA	AA	CC

^1^ Green are maintainer lines, red are restorer lines; light green and light red represent SNPs with a weaker confirmation by the KASP markers, white shows differences.

## Data Availability

Sequences used in this publication are given in Table 1 and the Supplementary Material (Table S5).

## Supplementary Materials

The following supporting information can be downloaded at: https://www.mdpi.com/article/10.3390/genes13030465/s1, Table S1: Lines (restorer and maintainer) and F1 hybrids with commercial tester lines; Table S2: Simulation of contaminations of restorer lines with different degrees of maintainer lines using hypocotyl pieces (1 cm) of 3-weeks-old sunflower seedlings; Table S3: Simulation of contaminations of restorer lines with different degrees of maintainer lines using leave pieces (Ø 1cm) of 3-weeks-old sunflower seedlings; Table S4: Transfer of the Gene IDs in the sunflower genome assembly HanXRQv1 to the new assembly HanXRQv2 for comparison of the localization of the SNPs in the potential restorer gene candidates; Table S5: Sequences around SNPs located in the three potential restorer genes.
