# Supplementary material for: KASP Markers Specific for the Fertility Restorer Locus Rf1 and Application for Genetic Purity Testing in Sunflowers (Helianthus annuus L.)
Source: Genes (Basel). 2022 Mar 5;13(3):465. doi: 10.3390/genes13030465 (PMC8951052; doi:10.3390/genes13030465)
Supplement: Supplementary file 1 [file genes-13-00465-s001.zip › genes-1611853-supplementary.pdf]

Supplementary Table S1: Maintainer lines (B), restorer lines (R) and F1-hybrids with commercial tester lines.

Accession ID	Common Name 1	Common Name 2	B/R	Hybrid *
Ames 31775	UGA-SAM1-082	ND-NONOIL B2	B	UGA-SAM1-082 x R
Ames 31801	UGA-SAM1-109	ND-EBLYS	B	UGA-SAM1-109 x R
Ames 31848	UGA-SAM1-156	HA 65	B	UGA-SAM1-156 x R
Ames 31877	UGA-SAM1-185	HA 821	B	UGA-SAM1-185 x R
CN36562	CM63	CM63	B	CM63 x R
CN36654	CM259	CM259	B	CM259 x R
PI 650649	Arrowhead	Arrowhead	B	Arrowhead x R
PI 221693	No. 2	No. 2	B	No. 2 x R
CN33280	Krasnodaret	Krasnodaret	B	-
CN42283	Armavirsky 3497	Armavirsky 3497	B	Armavirsky3497 x R
HRO	HA342	HA342	B	-
HRO	HA383	HA383	B	-
Ames 31703	UGA-SAM1-010	RHA 377	R	LC x UGA-SAM1-010
Ames 31717	UGA-SAM1-024	RHA 273	R	LC x UGA-SAM1-024
Ames 31792	UGA-SAM1-100	RHA 330	R	LC x UGA-SAM1-100
Ames 31793	UGA-SAM1-101	RHA 331	R	LC x UGA-SAM1-101
Ames 31813	UGA-SAM1-121	RHA 367	R	LC x UGA-SAM1-121
Ames 31828	UGA-SAM1-136	RHA 373	R	LC x UGA-SAM1-136
Ames 31853	UGA-SAM1-161	RHA 395	R	LC x UGA-SAM1-161
Ames 31861	UGA-SAM1-169	RHA 299	R	-
Ames 31883	UGA-SAM1-191	RHA 408	R	LC x UGA-SAM1-191
Ames 31896	UGA-SAM1-204	RHA 427	R	LC x UGA-SAM1-204
HRO	RHA325	RHA325	R	-
HRO	RHA265	RHA265	R	-
HRO	IH-51	IH-51	R	-
HRO	NS-H-27	NS-H-27	R	-

*R and LC represent commercial restorer and CMS tester lines, respectively

Supplementary Table S2: Simulation of contaminations of restorer lines (R) with different degrees of maintainer lines (B) using hypocotyl pieces (1 cm) of 3-weeks-old sunflower seedlings

%	0 % contamination	1% contamination	3% contamination	5% contamination	10% contamination	50% contamination
P	100 x 1 cm	99 x 1 cm (R)<br>1 x 1 cm (B)	97 x 1 cm (R)<br>3 x 1 cm (B)	95 x 1 cm (R)<br>5 x 1 cm (B)	90 x 1 cm (R)<br>10 x 1 cm (B)	50 x 1 cm (R)<br>50 x 1 cm (B)
1	UGA-SAM1-136 (R)	99 (R) + 1 x UGA-SAM1-109	97 (R) + 3 x UGA-SAM1-109	95 (R) + 5 x UGA-SAM1-109	90 (R) + 5 10 x UGA-SAM1-109	50 (R) + 50 x UGA-SAM1-109
2	UGA-SAM1-191 (R)	99 (R) + 1 x UGA-SAM1-109	97 (R) + 3 x UGA-SAM1-109	95 (R) + 5 x UGA-SAM1-109	90 (R) + 10 x UGA-SAM1-109	50 (R) + 50 x UGA-SAM1-109
3	UGA-SAM1-109 (B)	-	-	-	-	-
Σ	100	100	100	100	100	100

Supplementary Table S3: Simulation of contaminations of restorer lines (R) with different degrees of maintainer lines (B) using leaf discs (Ø 1cm) of 3-weeks-old sunflower seedlings

	0 % contamination	1% contamination	3% contamination	5% contamination	10% contamination	50% contamination
P	100 x 1 cm	99 x 1 (R) + 1 x 1 (B)	97 x 1 (R) + 3 x 1 (B)	95 x 1 (R) + 5 x 1 (B)	90 x 1 (R) + 10 x 1 (B)	50 x 1 (R) + 50 x 1 (B)
1	UGA-SAM1-136 (R)	99 (R) + 1 x UGA-SAM1-109	97 (R) + 3 x UGA-SAM1-109	95 (R) + 5 x UGA-SAM1-109	90 (R) + 10 x UGA-SAM1-109	50 (R) + 50 x UGA-SAM1-109
2	UGA-SAM1-191 (R)	99 (R) + 1 x UGA-SAM1-082	97 (R) + 3 x UGA-SAM1-082	95 (R) + 5 x UGA-SAM1-082	90 (R) + 10 x UGA-SAM1-082	50 (R) + 50 x UGA-SAM1-082
3	UGA-SAM1-109 (B)	-	-	-	-	-
4	UGA-SAM1-082 (B)	-	-	-	-	-
Σ	100	100	100	100	100	100

Table S4: Transfer of the Gene IDs in the sunflower genome assembly HanXRQv1 to the new assembly HanXRQv2r2 for comparison of the localisation of the SNPs in the potential restorer gene candidates

No	Annotation v1_gene	Annotation v2_gene	Annotation v2_position	Annotation v2_size	SNP-Name	Old position v1	New position v2	SNP	Gene Annotation
1	HanXRQChr13g0418841	HanXRQr2_Chr13g0609921	HanXRQChr13:155614426..155643618 (**- strand**)	29,193 bp	PPR841.26	170,851,288	155,642,485	T/C	Putative tetratricopeptide-like helical domain superfamily
2	HanXRQChr13g0418841	HanXRQr2_Chr13g0609921	HanXRQChr13:155614426..155643618 (**- strand**)	29,193 bp	PPR841.29	170,851,469	155,642,304	G/A	Putative tetratricopeptide-like helical domain superfamily
3	HanXRQChr13g0418841	HanXRQr2_Chr13g0609921	HanXRQChr13:155614426..155643618 (**- strand**)	29,193 bp	PPR841.38	170,851,758	155,642,015	G/A	Putative tetratricopeptide-like helical domain superfamily
4	HanXRQChr13g0418841	HanXRQr2_Chr13g0609921	HanXRQChr13:155614426..155643618 (**- strand**)	29,193 bp	PPR841.39	170,851,781	155,641,992	C/T	Putative tetratricopeptide-like helical domain superfamily
5	HanXRQChr13g0418861	HanXRQr2_Chr13g0609901	HanXRQChr13:155584507..155586558 (**- strand**)	2,052 bp	PPR861.3	170,906,233	155,587,499	G/A	Putative tetratricopeptide-like helical domain superfamily
6	HanXRQChr13g0418861	HanXRQr2_Chr13g0609901	HanXRQChr13:155584507..155586558 (**- strand**)	2,052 bp	PPR861.9	170,907,279	155,586,453	A/T	Putative tetratricopeptide-like helical domain superfamily
7	HanXRQChr13g0418861	HanXRQr2_Chr13g0609901	HanXRQChr13:155584507..155586558 (**- strand**)	2,052 bp	PPR861.11	170,907,603	155,586,129	C/T	Putative tetratricopeptide-like helical domain superfamily
8	HanXRQChr13g0418861	HanXRQr2_Chr13g0609901	HanXRQChr13:155584507..155586558 (**- strand**)	2,052 bp	PPR861.19	170,908,139	155,585,593	G/C	Putative tetratricopeptide-like helical domain superfamily
9	HanXRQChr13g0419621	HanXRQr2_Chr13g0608631	HanXRQChr13:152808601..152810798 (**+ strand**)	2,198 bp	PPR621.5	173,473,513	152,810,613	G/C	Putative tetratricopeptide-like helical domain superfamily, DYW domain-containing protein
10	HanXRQChr13g0419621	HanXRQr2_Chr13g0608631	HanXRQChr13:152808601..152810798 (**+ strand**)	2,198 bp	PPR621.11	173,473,976	152,810,150	C/A	Putative tetratricopeptide-like helical domain superfamily, DYW domain-containing protein

Table S5: Sequences around SNPs located in the three potential restorer genes

SNP-Name	SNP	Sequence position		HanXRQv1	HanXRQv2	Sequence area around SNPs
		Start	End	Orientation	Orientation	
PPR841.26	T/C	155,642,516	155,642,453	+	-	TACTTCTAAAGGTTTCGTCCCTAATGTTGTTACTTATAGCAGTTTACTGAA[T/C]GGGTATTGCAAGAGTTTGAAAATAGAAGAGGCCATGCATTTGTTTCATGA
PPR841.29	G/A	155,642,335	155,642,272	+	-	GGAGCTGCACGCAAATTCTTTGATGGGATGCGAGCACAAGGCCTTATTCCA[G/A]ATGAATGCACTTACGGAATAATATTAGATGGCCTTTGCAACAACCATCAA
PPR841.38	G/A	155,642,046	155,641,983	+	-	GTGGCTTGTGTGGGGAAGGTTTACTAAAGGAAGCAAAGCACTTGTTTCGTA[G/A]AATGGATGAGAGTGGCTGCCCACCAGATGGTGTTACTTACAATGTTCTTC
PPR841.39	C/T	155,642,023	155,641,960	+	-	CTAAAGGAAGCAAAGCACTTGTTTCGTAGAATGGATGAGAGTGGCTGCCCA[C/T]CAGATGGTGTTACTTACAATGTTCTTCTCCAGGGATATCTTAAGAAGCAG
PPR861.3	G/A	155,587,530	155,587,467	+	-	CAATTTCTCTTTTCCAAGACCTTACTGTAAAAGGTTTGAAACCTGATGTTC[G/A]GACACATAACGCAATGATTAGTGGCTTTTGTCGGAAGGGTCTGCTAAGAG
PPR861.9	A/T	155,586,484	155,586,421	+	-	CTTCTCCGTTGGAGGTTGCTGCGACAATGATGATGATTCGCACCAATTCTC[A/T]TCTTCATCACGCTTTCTTCAAACTCACAGGTATTCTCATCACCAATTCTT
PPR861.11	C/T	155,586,160	155,586,097	+	-	CCAAAATGAAACATTTTTCTTGCTCTCTTGACCTTTTCAAACAAATGTGTG[C/T]CATTGGAGTTCCTGTTGACAAATACTCTATGAGTATTGCCATCAAGTGTT
PPR861.19	G/C	155,585,624	155,585,561	+	-	TTATGTCGTTGGGACGAGGTCTCTAAGCTGCTAAAAGAAATGGAGGAGGAT[G/C]TAAGGATCTCTCCTAATATTCAAACCTTTAGCATATTAGTTGATGCATTT
PPR621.5	G/C	152,810,644	152,810,581	-	+	CGGGAGATTATCGTTCGCGATGCTAGTAGATTTCATCATTTTAAAGATGGT[C/G]AATGTTCATGTGGAGATTACTGGTAAGATGTTATAATTATTATTCAATTA
PPR621.11	C/A	152,810,181	152,810,118	-	+	AGAAGTCATACGGGTGGACCCACATGATTCAGCATCATACGTTTTACTATC[G/T]AACATACAAGCGTCCGCTAAAAAATGGCACGATGTGTCGGATTTCCGAAA
